# A dietary risk assessment of the pyrethroid insecticide resmethrin associated with its use for West Nile Virus mosquito vector control in California

**DOI:** 10.1100/tsw.2006.56

**Published:** 2006-02-27

**Authors:** Wesley C. Carr, Poorni Iyer, Derek W. Gammon

**Affiliations:** ^1^ California EPA, Department of Pesticide Regulation, Medical Toxicology Branch, Sacramento, CA, USA; ^2^ California EPA, Office of Environmental Health Hazard Assessment, Sacramento, CA, USA

**Keywords:** West Nile Virus, WNV, encephalitis, meningitis, resmethrin, pyrethroid, mosquito, dietary exposure

## Abstract

An outbreak of human illnesses associated with West Nile Virus (WNV) occurred in New York City in 1999. Since then, it has gradually spread westwards, reaching northern California for the first time in 2005. WNV is transmitted by several mosquito species and birds serve as the main reservoir. Several control measures have been used, targeting both the aquatic larvae and the adult mosquitoes. In the latter case, roosting birds in trees are sprayed with pyrethroid insecticides because these are highly toxic to mosquitoes, but have low avian toxicity. A request was made to use a resmethrin-containing insecticide during the month of October 2005 in California. Because resmethrin was not registered for use on growing crops, concerns were raised about potential crop contamination. Therefore, an expedited dietary risk assessment was conducted on resmethrin. Developmental toxicity in the rat (NOELs of 25 or 40 mg/kg/day) was used as the acute endpoint and dietary exposure was assessed using the DEEM-FCID^TM^ computer program. Only crops growing above ground during October were considered. Margins of Safety (MOS) were found to be above 100, the level generally considered to be sufficient to protect public health when using an animal NOEL.

## INTRODUCTION

West Nile Virus (WNV) is an arbovirus that is transmitted by a number of mosquito species[1]. It can cause both neuroinvasive and non-neuroinvasive diseases in birds and mammals, including humans. Both types of disease in humans are associated with fever and neuroinvasive diseases can include encephalitis, meningitis, and myelitis. In severe cases, death or permanent neurological impairment may result. Cases of encephalitis from infection by WNV first appeared in the U.S. in 1999, in New York City[2]. WNV-associated diseases have spread westward since 1999, and in 2005, first affected northern California.

Note: The opinions expressed are the authors' and do not necessarily reflect the policies of Cal/EPA.

During 2005, there were 873 human cases in California, 294 of which involved neuroinvasive disease, with 18 deaths. Within California, Sacramento County had the largest number (177) of these cases[3]. In the U.S. as a whole, there were 2,949 cases in 2005, 1,272 of which involved neuroinvasive diseases, along with 116 deaths. Human cases were found in 42 of the 48 contiguous states. Most of the deaths occurred in June, July, and August. Although a vaccine exists for horses, one of the main control measures to avoid human infection is control of the mosquito vectors. Both the aquatic larvae and the adults have been targeted. In the latter case, the measures have included aerial spraying of roosting birds, the primary reservoirs of WNV, with pyrethroid insecticides.

The California Department of Health Services (DHS) requested a Section 18 Emergency Exemption public health registration for the Bayer Crop Science product Scourge® to control WNV-carrying mosquito vectors. Public health applications of pesticides to mosquito adults generally involve spraying birds in trees during the evening and early morning hours, so as to reduce the mosquito population. The possibility was raised that Scourge® could inadvertently be sprayed above food crops or could drift onto them during such an application. Scourge® contains the active ingredients resmethrin (CAS# 10453-86-8) and piperonyl butoxide or PBO (CAS# 51-03-6) at 18 and 54%, respectively. The latter is not insecticidal, but acts as a synergist by slowing down the oxidative metabolism of resmethrin in insects. An estimated dietary exposure assessment for resmethrin, the insecticidal component, was therefore conducted by the Department of Pesticide Regulation (DPR) and the toxicity of PBO was also taken into consideration.

Resmethrin, [5-(phenylmethyl)-3-furanyl] methyl 2,2-dimethyl-3-(2-methyl-1-propenyl) cyclopropane-carboxylate, is on the California Proposition 65 list[4]. Such chemicals are listed when they are “known to the State to cause cancer or reproductive toxicity.” Warnings to the general public need to be posted whenever there exists the possibility of the chemical entering any source of drinking water. In practice, this means “exposure at a level 1,000 times greater than the maximum allowable dose level is expected to have no observable effect.” Resmethrin was listed in 1994 through the “Authoritative Bodies” mechanism because of developmental toxicity reported by theUSEPA[4], in a rat reproductive toxicity study.

Currently, there are 51 pesticide products containing resmethrin registered in California by the DPR[5]. Based on the most recently published DPR 2003 Pesticide Use Report (PUR), only 1,561 lb of resmethrin were applied in California during 2003[6]. The two main uses were for public health and commercial applications for structural pest control. These uses accounted for approximately 94% of total use reported in the California PUR. There are no established or proposed USEPA raw agricultural commodity tolerances (also known as MRLs or maximum residue limits) for resmethrin[7]. The only existing tolerance is for indirect residues in or on food items in food handling and storage areas. This 3-ppm tolerance is for residues that may result from crack and crevice and/or spot treatments from liquid formulations[7]. A USEPA Reregistration Eligibility Document (RED) for resmethrin is not scheduled for completion until May 2006.

The purpose of this study was to analyze the environmental fate and toxicology databases for suitable studies and endpoints for risk assessment of resmethrin. Subsequently, a theoretical dietary exposure analysis was conducted using DEEM-FCID™[8], concentrating especially on population subgroups likely to be highly exposed or most at risk. Finally, margins of safety (MOS) were calculated for these groups.

## CHEMISTRY/ENVIRONMENTAL FATE

The Type I pyrethroid (see below) resmethrin is a racemic mixture of four isomers. It is insoluble in water (<1 mg/l) and ranges from soluble to very soluble in various organic solvents (methanol, 81 g/l; hexane, 220 g/l; and xylene, >1 kg/l)[9]. While mostly insoluble in water, resmethrin is still very toxic to fish with 96-h $LC_{50}$ values ranging between 0.3–5.5 µg/l[9]. The vapor pressure of resmethrin is very low ($1.1 \times 10^{-8}$ mmHg at 30°C[6]; <0.01 mPa [10]). The half-life ($t_{1/2}$) of the toxicologically relevant parent resmethrin in the natural environment is relatively short. Similar to several other Type I pyrethroids (e.g., allethrin, phenothrin), resmethrin contains a chrysanthemic acid moiety that is susceptible to breakdown from ultraviolet wavelength solar radiation, microbial, animal and plant metabolic degradation[1,9]. Oxidation and ester cleavage are the primary processes. The photodegradation $t_{1/2}$ of resmethrin in distilled water ranges between 31–47 min[1,9,11]. Soil degradation is also fairly rapid, with only approximately 2% of the parent material remaining after 16 days[9]. A registrant submitted soil photodegradation study showed a $t_{1/2}$ of 15.4 h[10]. Plant degradation following experimental agronomic applications is rapid with no residues detectable after 5 days due to ester cleavage and breakdown of the chrysanthemic acid moiety by UV radiation[9].

## TOXICOLOGY

Resmethrin is classed as a Type I pyrethroid, acting on voltage-sensitive sodium channels in insect nerve axons, resulting in repetitive firing following stimulation[12]. In mammals, it causes clinical signs that have been called the Type I or T (tremors) syndrome[13]. Mammals and birds are less susceptible than insects to the toxicity of Type I pyrethroids, including resmethrin, for a variety of reasons, including more rapid metabolic breakdown (in mammals)[10,13]. A toxicology database is available to the DPR that describes studies conducted in support of registration requirements. Studies have been conducted, on behalf of the registrants, using laboratory animals to define both the short-term (acute) effects as well as the long-term (chronic) effects, including cancer. For the purposes of the limited duration of Scourge® use in California, developmental toxicity was considered to be the most appropriate type of study to use for acute dietary risk assessment. Although such studies involve multiple maternal dosing, it is considered that developmental toxicity may result from a single day's exposure during gestation. In order to be health protective, each day of gestation is considered to offer a potential novel window of sensitivity of the fetus for the chemical.

### Developmental Toxicity

For assessing acute toxicity, developmental toxicity studies are often the most sensitive (lowest no-observed-effect level [NOEL]) and there are seven such studies in the DPR SB950 database (rat, four; rabbit, two; mouse, one). Additionally, there are three rat reproduction studies available. There is no rat acute oral neurotoxicity study available in the DPR database because it is not currently a required study for the synthetic pyrethroid class of insecticides.

Only one of four rat teratology studies was considered acceptable and useful for risk assessment. In this single acceptable study[14], 25 pregnant SD rats per dose were dosed by oral gavage with resmethrin at 0, 20, 40, or 80 mg/kg/day on gestation day (GD) 6–15 and sacrificed on day 20 (Table 1). The significant effects were reduced maternal body weight at GD 20 ($p < 0.05$) and reduced food consumption (GD 11–15, $p < 0.05$). The only significant developmental effect of resmethrin was delayed ossification in the skull of fetuses at 80 mg/kg/day, the LOEL (lowest observed effect level). The maternal and fetal NOEL was therefore 40 mg/kg/day. The NOEL from this study was used to estimate dietary risk.

Two studies[15,16] were unacceptable for several reasons and the fourth study was a review of multiple pesticides including resmethrin[17]. According to this review, resmethrin was administered by oral gavage to pregnant rats at 10, 20, and 50 mg/kg/day, on GD 9–14, without any significant adverse effects. Similarly, it was administered to mice at 10, 30, and 50 mg/kg/day, on GD 7–12, also without adverse effects. No data were provided.

There were two rabbit teratology studies submitted to the DPR, of which one was acceptable to the DPR and the other was seriously flawed and considered unacceptable and not upgradeable. In the acceptable study, groups of 18 pregnant New Zealand White rabbits were dosed orally by gavage with resmethrin in corn oil at 0 (vehicle), 0 (sham), 30, 120, or 300 mg/kg/day on GD 6–18 and sacrificed on day 29[18]. Maternal toxicity (Table 2) was observed only at 300 mg/kg/day in the form of reductions in body weight (*p*

**TABLE 1**
**Developmental and Maternal Toxicity of Resmethrin in the Rat**

| | 0 | 20 mg/kg/day | 40 mg/kg/day | 80 mg/kg/day |
|---|---|---|---|---|
| **Maternal effects** | | | | |
| Mean body wt.GD 20, g (n) | 377 ± 9.3 (25) | 372 ± 6.5 (23)<br>1.3%↓ | 358 ± 10.4 (23)<br>5.0%↓ | 330 ± 16.0* (21)<br>12.5%↓ |
| Food consumed GD 6–11, g | 76.5 ± 9.2 | 65.2 ± 3.1<br>14.8%↓ | 58.6 ± 4.1<br>23.4%↓ | 60.8 ± 4.2<br>20.5%↓ |
| Food consumed GD 11–15 | 65.6 ± 3.2 | 53.5 ± 3.9*<br>18.4%↓ | 47.9 ± 4.2*<br>27.0%↓ | 50.5 ± 4.6*<br>23.0%↓ |
| **Developmental effects** | | | | |
| Skeletal anomaly, skull | | | | |
| Litter data | 3/23 (13%) | 1/22 (4.6%) | 2/22 (9.1%) | 4/17 (24%) |
| Fetal data | 3/187 (1.6%) | 1/161 (0.6%) | 2/166 (1.2%) | 11/136** (8.1%) |

* $p < 0.05$.
** $p < 0.01$ (Fisher's exact test).

**TABLE 2**
**Developmental Toxicity of Resmethrin in the New Zealand White Rabbit[1]**

| Effect | 0 (Vehicle) | 0 (Sham) | 30 mg/kg/day | 120 mg/kg/day | 300 mg/kg/day |
|---|---|---|---|---|---|
| **Maternal effects** | | | | | |
| Body wt. (kg)<br>Day 19 (n) | 3.88 ± 0.29 (18) | 3.94 ± 0.30 (18) | 3.84 ± 0.23 (18)<br>(1.0%↓) | 3.84 ± 0.37 (18)<br>(1.0%↓) | 3.58 ± 0.48* (18)<br>(7.7%↓) |
| Body wt. change, kg<br>Day 6–19 | 0.23 ± 0.11 | 0.23 ± 0.07 | 0.20 ± 0.12 | 0.16 ± 0.13 | –0.04 ± 0.34* |
| Food eaten, g/day<br>Day 6–19 | 158 ± 28 | 176 ± 11 | 153 ± 31<br>(3.2%↓) | 144 ± 32<br>(8.9%↓) | 109 ± 50**<br>(31%↓) |
| Food eaten, g/kg<br>Body wt./day<br>Day 6–19 | 41.8±6.5 | 46.2 ± 4.5 | 40.7 ± 7.8<br>(2.6%↓) | 38.0 ± 6.5<br>(9.1%↓) | 29.5 ± 12.4**<br>(29%↓) |
| Dry/no feces<br>Day 6–29 | 0 | 0 | 0 | 2/5 | 6/26**[,2] |
| **Developmental effects** | | | | | |
| Abortions | 0/18 | 0/15 | 0/15 | 0/13 | 5/17**[,3] (29%) |
| Complete resorptions | 0/18 | 0/15 | 0/15 | 0/15 | 2/17** (12%) |
| Skull: irregular ossification: | | | | | |
| Litter data | 15/18 (83%) | 14/15 (93%) | 15/15 (100%) | 11/13 (85%) | 9/10 (90%) |
| Fetal data | 44/136 (32%) | 50/109 (46%) | 48/108 (44%) | 28/97 (29%) | 33/81 (41%)* |
| Fetal alteration: | | | | | |
| Litter data | 18/18 (100%) | 15/15 (100%) | 15/15 (100%) | 13/13 (100%) | 10/10 (100%) |
| Fetal data | 66/136 (49%) | 61/109 (56%) | 63/108 (58%)* | 36/97 (37%)** | 42/81 (52%) |

1 Data from [18]; does were dosed daily by oral gavage on GD 6–18.
2 6 cases were noted out of 26 observations; there were 4 does/18 showing this effect.
3 4 of these (5) does were those with dry or no feces; they aborted on GD 21, 24, 24, and 26.
* $p < 0.05$.
** $p < 0.01$.

$< 0.05$), body weight gain ($p < 0.05$) and food intake ($p < 0.01$). There was also an increase ($p < 0.01$) in the incidence of does with dry or no feces at this dose. A total of five does (300 mg/kg/day dose group) had abortions. Four of these individuals also had the dry or no feces for 2–6 days preceding the abortion. The no or dried feces effect was also reported in two does in the 120 mg/kg/day dose group. Another developmental effect observed at 300 mg/kg/day was complete resorption of 2 of 17 litters ($p < 0.01$). There were no dose-related increases in fetal visceral or skeletal abnormalities. The maternal and developmental LOEL/NOEL in the rabbit were therefore 300 and 120 mg/kg/day, respectively.

In an unacceptable study[19], groups of 20 pregnant rabbits (hybrid mixture of New Zealand Whites) were dosed by gavage with resmethrin at 0, 10, 30, or 100 mg/kg/day on GD 6–18 and sacrificed on day 29. There were no reported treatment-related maternal effects. However, reproductive/developmental effects included increased extra sternebrae at 100 mg/kg and increased resorptions, dark livers, and fused sternebrae at 10, 30, and 100 mg/kg. A large number of testing protocol problems associated with this study precluded its use for risk assessment.

## Reproductive Toxicity

From the three available multiple-generation rat reproduction studies, the only acceptable study was used to estimate theoretical dietary risk for females of childbearing age. The 1978 study[20] was not used since it followed only a single generation. Similarly, a rat three-generation study was severely compromised by numerous deficiencies. The only DPR-acceptable study was a two-generation study[21].

In this study, groups of 30 Crl:CDBRVAF/Plus rats/sex/dose were fed diets containing resmethrin at 0, 250 (12.5 mg/kg/day), 500 (25 mg/kg/day), and 1,000 ppm (50 mg/kg/day) through two generations (F0, F1) with two litters/generation (Table 3). Treatment-related effects recorded at 1,000 ppm (50 mg/kg/day) were decreased pup viability (F1a, F1b, F2a, F2b litters), increased dead pups on day 1 ($p < 0.01$), transient decrease in F1a and F2a pup body weights, $p < 0.01$ (day 1), decreased fertility in F2b mating, and decreased food consumption by F0 females during nursing, probably related to reduced litter size (Table 3). Although two F1b males died (6.7%) on days 37 and 44 postweaning, at 1,000 ppm, the incidence was not statistically different from control mortality (0%). There was no significant reduction in maternal body weight or body weight gain. The NOEL for adults was therefore 1,000 ppm, and for pups was 500 ppm (25 mg/kg/day) based on effects described at 1,000 ppm (50 mg/kg/day). The NOEL from this study was used to estimate dietary risk.

In a pre-GLP (Good Laboratory Practices) dietary study, groups of 30 rats/sex/dose were fed resmethrin at 0, 500 (25 mg/kg/day), 1,000 ppm (50 mg/kg/day), and 1,250 ppm (62.5 mg/kg/day) through three generations[22]. No chronic toxicity was reported in the adult animals. For the pups, treatment-related reproductive toxicity was reported in all dosed groups. The effects were reduced litter size, and decreased pup survival and average body weights. The LOEL for the study was 25 mg/kg/day (500 ppm in the diet), but the study was considered too flawed to be used for risk assessment.

From the previously discussed toxicity studies, two scenarios were selected to quantify the potential acute dietary risk from theoretical residues of resmethrin when applied in or on agricultural crops during public health mosquito vector control. The NOEL of 40 mg/kg/day, based on delayed fetal ossification and maternal effects, from the rat oral gavage teratology study[14] was used to estimate acute dietary risk for all populations except females of childbearing age (Table 1). For this population subgroup, the NOEL of 25 mg/kg/day (500 ppm in the diet) based on developmental toxicity in the rat two-generation dietary reproductive toxicity study[21] was used to estimate potential acute dietary risk (Table 3). This NOEL is based on statistically significantly decreased pup viability and day 1 mean pup body weight measurements reported at 50 mg/kg/day (1,000 ppm in the diet).

**TABLE 3**
**Reproductive Toxicity of Resmethrin in the Rat in a Two-Generation Study**

| Generation/Effect | 0 | 250 | 500 | 1,000 ppm |
|---|---|---|---|---|
| **F0** | | | | |
| Maternal wt, GD20 (n) | 386 ± 26 (28) | 389 ± 28 (25) | 389 ± 27 (28) | 379 ± 26 (27) |
| Maternal wt gain, 0–20 | +115 ± 17 | +115 ± 16 | +118 ± 13 | +109.5 ± 23 |
| **F1a litters** | | | | |
| Viability index D1-4 | 98.5% | 97.7% | 96.7% | 66.5%** |
| Still born | 2 (0.5%) | 3 (0.8%) | 9 (2.2%) | 12 (3.4%) |
| Dead on D1 | 2 | 3 | 3 | 13** |
| Total No. fetuses | 400 | 397 | 408 | 355 |
| Mean Pup wt. D1 | 6.1 ± 0.6 | 5.9 ± 0.5 | 5.8 ± 0.3 | 5.6 ± 0.5** (8.2%↓) |
| Mean No. live pups/litt D4 | 13.5 ± 2.6 | 13.3 ± 2.4 | 13.8 ± 1.6 | 8.4 ± 8.4** (38%↓) |
| **F1a dams** | | | | |
| Maternal wt, GD20 (n) | 422 ± 35 (24) | 434 ± 30 (23) | 422 ± 38 (22) | 430 ± 32 (22) |
| Maternal wt gain, 0-20 | +115 ± 24 | +119 ± 20 | +113 ± 25 | +116 ± 19 |
| **F1b litters** | | | | |
| Viability index D1-4 | 96.6% | 98.1 | 98.1 | 81.8%** |
| Still born | 4 (1.2%) | 3 (0.9%) | 1 (0.4%) | 5 (1.8%) |
| Dead on D1 | 4 | 2 | 2 | 13** |
| Total No. fetuses | 324 | 327 | 268 | 282 |
| Mean Pup wt. D1 | 6.1 ± 0.8 | 6.0 ± 0.6 | 6.2 ± 0.7 | 5.7 ± 0.5 (6.6%↓) |
| Mean No. live pups/litt D4 | 12.9 ± 3.3 | 13.8 ± 2.4 | 11.8 ± 4.2 | 10.2 ± 5.0 (21%↓) |
| **F1b dams** | | | | |
| Maternal wt, GD20 (n) | 389 ± 34 (21) | 400 ± 35 (24) | 405 ± 44 (23) | 388 ± 36 (24) |
| Maternal wt gain, 0–20 | +108 ± 21 | +111 ± 15 | +114 ± 21 | +108 ± 15 |
| **F2a litters** | | | | |
| Viability index D1-4 | 99% | 94.1% | 95.3% | 54.4%** |
| Still born | 2 (0.7%) | 5 (1.4%) | 1 (0.3%) | 6 (1.8%) |
| Dead on D1 | 0 | 9* | 4 | 15** |
| Total No. fetuses | 297 | 363 | 302 | 342 |
| Mean Pup wt. D1 | 6.0 ± 0.6 | 6.0 ± 0.8 | 5.8 ± 0.4 | 5.5 ± 0.5** (8.3%↓) |
| Mean No. live pups/litt D4 | 12.6 ± 3.8 | 12.5 ± 3.6 | 12.4 ± 2.7 | 7.2 ± 5.0** (43%↓) |
| **F1b dams** | | | | |
| Maternal wt, GD20 (n) | 431 ± 58 (24) | 442 ± 41 (23) | 439 ± 48 (26) | 424 ± 37 (18) |
| Maternal wt gain, 0–20 | +108 ± 27 | +112 ± 20 | +106 ± 26 | +108 ± 14 |
| **F2b litters** | | | | |
| Viability index D1-4 | 92.6% | 99.0% | 94.6% | 84.1%** |
| Still born | 3 (0.9%) | 4 (1.4%) | 12** (3.9%) | 2 (0.8%) |
| Dead on D1 | 1 | 0 | 4 | 5** |
| Total No. fetuses | 340 | 293 | 324 | 235 |
| Mean Pup wt. D1 | 5.9 ± 0.9 | 6.1 ± 0.6 | 6.2 ± 0.7 | 5.6 ± 0.5 (5.1%↓) |
| Mean No. live pups/litt D4 | 13.0 ± 4.6 | 12.3 ± 2.8 | 10.7 ± 4.2* | 11.5 ± 2.7* (12%↓) |

* $p < 0.05$.
** $p < 0.01$.

## Other Toxicological Issues

In other guideline studies, submitted under the Federal Insecticide Fungicide & Rodenticide Act (FIFRA), the chronic toxicity of resmethrin was assessed in the rat, mouse, and dog. In a 2-year dietary study, oncogenicity was negative in the rat, but there was some evidence for liver tumors in the mouse (Table 4). These were both hepatocellular adenomas and (malignant) carcinomas[23]. The increased incidence was dose dependent and statistically significant at all three dose levels (300, 600, and 1,200 ppm), but was restricted to males.

**TABLE 4**
**Chronic Toxicity of Resmethrin in the Mouse[1]**

| Effect | 0 (A) | 0 (B) | 300 | 600 | 1,200 ppm |
|---|---|---|---|---|---|
| **Males** | | | | | |
| Mean liver wt., g | 1.4 ± 0.2 | 1.9 ± 1.0 | 2.1 ± 1.0 | 2.2 ± 0.9 | 2.7 ± 1.1 |
| Diffuse hepatocyte hypertrophy | 3/50[2] (6%) | 0/50 (0%) | 6/50 (12%) | 5/50 (10%) | 19/50*** (38%) |
| Centrilobular hep. hypertrophy | 0[3] (0%) | 0[3] (0%) | 6/50* (12%) | 9/50** (18%) | 18/50*** (36%) |
| Hepatocellular adenoma (B) | 1/50[4] (2%) | 8/50 (16%) | 9/50* (18%) | 10/50* (20%) | 12/50*** (24%) |
| Hepatocellular carcinoma (M*) | 2/50[5] (4%) | 0/50 (0%) | 2/50 (4%) | 4/50 (8%) | 6/50* (12%) |
| Hepatocellular tumors, M* plus B | 3/50[6] (6%) | 8/50 (16%) | 11/50* (22%) | 14/50** (28%) | 18/50*** (36%) |
| **Females** | | | | | |
| Mean liver wt., g | 1.4 ± 0.3 | 1.3 ± 0.3 | 1.5 ± 0.5 | 1.8 ± 0.7 | 2.0 ± 0.6 |
| Diffuse hepatocyte hypertrophy | 2/50[7] (4%) | 0/50 (0%) | 1/50 (2%) | 5/50* (10%) | 8/50** (16%) |
| Centrilobular hep. hypertrophy | 0/50(0%) | 0/50(0%) | 0/50(0%) | 0/50(0%) | 0/50(0%) |
| Hepatocellular adenoma (B) | 2/50 (4%) | 0/50 (0%) | 1/50 (2%) | 2/50 (4%) | 3/50 (6%) |
| Hepatocellular carcinoma (M*) | 0/50 (0%) | 0/50 (0%) | 0/50 (0%) | 0/50 (0%) | 1/50 (2%) |
| Hepatocellular tumors, M* plus B | 2/50 (4%) | 0/50 (0%) | 1/50 (2%) | 2/50 (4%) | 4/50 (8%) |

1 Data from [23].
2 ****p* < 0.001 (using control A); *p* < 0.05, *p* < 0.05, and *p* < 0.001 (using Control B) for the three dose groups, respectively (Fisher's exact test) *p<0.05, **p<0.01.
3 *p* < 0.05, *p* < 0.01, and *p* < 0.001 (Control A or B) for the three groups, respectively.
4 *p* < 0.05, *p* < 0.05, and *p* < 0.001 (Control A or N.S. for B) for the three groups, respectively.
5 N.S. Control A or *p* < 0.05, for B.
6 *p* < 0.05, *p* < 0.01, and *p* < 0.001 (Control A) for the three groups, respectively, and *p* < 0.05 at 1,200 ppm (Control B).
7 *p* < 0.05 (Control A) at the top dose and *p* < 0.05 or *p* < 0.01 (Control B) for the top two doses, respectively.

Moreover, there was evidence of significant hepatocyte hypertrophy (diffuse and especially centrilobular), which also showed a dose response (Table 4). Such hypertrophy is common for hepatic enzyme inducers and is often considered to be a precancerous condition. However, for structural analogs such as the Type I pyrethroid pyrethrin, such hypertrophy demonstrated a threshold, and dissipated after the discontinuation of dosing[24]. It should be noted that for females, which did not show an elevated incidence of liver tumors with resmethrin, there was little evidence of hepatocyte hypertrophy compared with males (Table 4). Furthermore, no genotoxicity was observed in experiments designed to measure gene mutation, chromosome aberrations, and DNA damage. It must therefore be concluded that the liver tumors resulting from resmethrin dosing in the mouse have little relevance for human cancer risk assessment.

The synergist PBO has recently been the subject of a reregistration eligibility document[25]. In addition to acting as an inhibitor of the MFOs (Mixed Function Oxygenase(s)) responsible for remethrin metabolism, PBO has been shown to also induce liver enzymes. Tumors were observed in lifetime feeding studies in the mouse (liver), though not in the rat. However, these were significantly elevated only at high doses, they were benign and were associated with liver toxicity. It was demonstrated that liver tumors were increased only at doses causing liver toxicity. Also, some of the genes induced in liver (MFOs) by PBO were identified. Genotoxicity assays with PBO were negative. It was concluded that liver tumors show a clear threshold and are secondary to liver toxicity (enzyme induction) with little, if any, human relevance[25]. In other experiments, PBO did not cause reproductive toxicity in a two-generation study in the SD rat, even at doses causing developmental toxicity, in the form of reduced pup body weight (250 mg/kg/day). In developmental toxicity studies in the rat and rabbit, where PBO was administered by oral gavage on gestation days 6–15 and 7–19, respectively, only slight effects on the fetus were observed at doses causing clear maternal toxicity.

## EXPOSURE

### Residue Levels in Commodities

An acute dietary exposure assessment was conducted to represent theoretical risk from estimated commodity residues resulting from public health applications of resmethrin to control WNV mosquitoes. There are no established USEPA resmethrin tolerances (or exemptions) for Raw Agricultural Commodities (RACs). Therefore, any residues detected on RACs would constitute a no-tolerance-established (NTE) enforcement issue and would result in the impounding of a grower's affected commodities. However, the likelihood of detectable residues in the harvested commodities would be small due to the public health vector control program's lower treatment rate (0.007 lb a.i./acre maximum) and higher aerial application height above crop fields when compared to conventional agricultural practices. There is an exemption from USEPA tolerances for RACs treated with the structurally similar Type I pyrethroid allethrin[26] when applied prior to harvest under normal agricultural conditions. A total of 43 RACs are covered by the allethrin tolerance exemption including infant and children high-consumption commodities such as apple, citrus crop group, peach, pear, potato, and tomato. This is likely indicative of the expectation by the USEPA of no detectable residues remaining in the crops following harvest.

A total of 157 commodities were included in the DPR theoretical estimated residue dietary commodity file for resmethrin. Only California-grown commodities that were likely to be harvested during the October 1 – November 1 Section 18 use period were considered[27]. Since there is no systemic activity associated with resmethrin, tubers would not likely have any potential residues. Hence, edible below-ground commodities (e.g., tubers), while harvested during this period, were deleted from the dietary file. Therefore, only edible fruits, leaves, nuts, and stalks of harvestable commodities were included in the analysis. No drinking water was included in the commodity residue file for three reasons: resmethrin is broken down very rapidly in water, it is not anticipated to be used near water, and because it readily binds to solids rather than staying in solution (discussed earlier). The 3-ppm resmethin food residue tolerance for indirect residues in food handling and storage areas resulting from crack and crevice and/or spot treatments was used to represent almost all of the commodities included in the analysis. Estimated theoretical residues for rice grain and alfalfa seed RACs were 1 and 2 ppm, respectively, based on field studies on these crops with resmethrin[11].

### Acute Theoretical Dietary Exposures

The DPR resmethrin assessment used the DEEM-FCID™ acute dietary exposure software[8]. The DEEM-FCID™ analysis program uses the 1994–98 USDA Continuing-Survey-of-Food-Intake-by-Individuals (CSFII) food survey[28]. This survey provides the most extensive dietary consumption information available for the U.S. population. Exposure was calculated using estimated deterministic theoretical commodity values, default food forms, and percent crop treated adjustment factors (Tier 1 analysis). The deterministic exposure was calculated using the entire distribution of a single day's food consumption and then combined with a single residue level for each commodity to generate a distribution of possible exposure (in mg/kg/day). Two acute endpoints were used in the assessment: one to represent the general population subgroups and one to represent females of childbearing age. The general population dietary endpoint includes the infants and children subgroups. This exposure was compared with the NOEL of 40 mg/kg/day from the rat acute oral gavage teratology study[14]. For females of childbearing age, the 25 mg/kg/day NOEL (500 ppm in the diet) from the rat two-generation dietary reproductive study[21] was used to calculate a MOS from possible reproductive and/or teratogenic effects following acute exposure (Table 5). The MOS is defined as the NOEL divided by the estimated exposure. A MOS value above 100 is generally considered to be adequate to protect human health, whenever the NOEL is from an animal study.

**TABLE 5**
**Summary of Theoretical Dietary Exposure and Risk for Resmethrin**

| Population Subgroup | Acute Dietary [1] (97.5th Percentile) | |
|---|---|---|
| | Dietary Exposure[3,4] (mg/kg/day) | MOS |
| U.S. population (all seasons) | 0.083 | 480 |
| All infants (<1 year old) | 0.191 | 210 |
| Children 1–2 years old | 0.218 | 180 |
| Children 3–5 years old | 0.159 | 250 |
| Children 6–12 years old | 0.092 | 430 |
| Hispanics | 0.095 | 420 |
| Non-Hispanic Whites | 0.078 | 510 |
| Non-Hispanic Blacks | 0.093 | 430 |
| Males 20+ years | 0.043 | 930 |
| **Females 13–49 years old[2]** | **0.048** | **520** |

1 Acute dietary endpoint of 40.0 mg/kg/day (Table 1) applies to the general U.S. population (including infants and children population subgroups). Excludes the females 13–49 years old group.

2 Acute dietary endpoint of 25.0 mg/kg/day (Table 3) applies to females 13–49 years old only. The MOS is 830 using the NOEL of 40 mg/kg/day.

3 Alfalfa seed, almond, apple, artichoke (globe and Jerusalem), avocado, basil, beans (black, broad, cowpea, great northern, kidney, lima, mung, navy, pink, pinto, and snap), garden beet tops, Belgium endive, blueberry, broccoli, Brussels sprouts, cabbage, cantaloupe, carrot, casaba, cauliflower, celeriac, celery, chickpea, Chinese waxgourd, chive, collards, coriander, sweet corn, crabapple, cress, cucumber, dandelion leaves, date, dillweed, eggplant, endive, fennel, gooseberry, grape, grapefruit, herbs, honeydew, kale, kiwifruit, kohlrabi, lemon, lentil, lettuce, mustard greens, okra, olive, orange (Valencia), parsley leaves, pea (succulent and pigeon), pear, pepper (bell and nonbell), peppermint, persimmon, pistachio, pomegranate, pumpkin, quince, radicchio, radish tops, rape greens, rice, salsify tops, savory, spearmint, spices, spinach, squash (winter), strawberry, Swiss chard, tomatillo, tomato, turmeric, turnip greens, walnut, watercress, watermelon, and wild rice.

4 Consumption based on user-day exposure, where a user-day is defined as any day on which any one or more of the commodities in (3), above, was consumed. Percent user-days for all population subgroups were >99%, with the exception of nursing infants (52%) and non-nursing infants (83%).

The acute MOS at the 97.5th percentile of exposure ranged from 180 for children 1–2 years to 930 for males 20+ years when a NOEL of 40 mg/kg/day and the 1994-98 CSFII data were used (Table 5). When using the same commodities and estimated residue values, the MOS for females in the 13- to 49-year-old population subgroup at the 97.5th percentile of exposure is 520 when the NOEL of 25 mg/kg/day was used (Table 5).

## DISCUSSION

The use of resmethrin above agricultural fields as a result of the public health Emergency Exemption petition is unlikely to result in measurable crop residues, due to remethrin's rapid chemical and biological degradation under field conditions and the low application rates proposed. However, there are no current RAC USEPA tolerances for resmethrin. The USEPA would need to be consulted regarding obtaining an exemption from tolerance for resmethrin resulting from public health vector control similar to the agricultural RAC exemptions that exist for allethrin. Any detectable residues recovered on commodities resulting from the public health mosquito control program would constitute a NTE illegal residue enforcement issue. However, for a registered pesticide/RAC combination, residues are generally considered to have negligible toxicity when the MOS > 100, whenever the critical NOEL is based on animal studies, as here.

The temporary registration of a resmethrin plus PBO insecticide for aerial spraying against mosquitoes, the vectors of WNV encephalitis, involved the analysis of the toxicology databases and literature for information on these compounds. Developmental toxicity was chosen as the critical endpoint since this was the most sensitive measure for acute effects of possible short-term exposure. Using such endpoints, from a rat study, in which dams were dosed by oral gavage on a daily basis throughout the critical period of gestation (during which organs are formed in the fetus), represents a conservative assessment of toxicity for most population subgroups. It is also possible, for example, that the delayed fetal ossification that was observed at the LOEL (80 mg/kg/day), though not at the NOEL (40 mg/kg/day), was secondary to maternal toxicity (reduced mean body weight and food consumed), which was also found at this LOEL. It should also be noted that developmental toxicity was statistically significant ($p < 0.01$) only at the fetal level and not at the (preferred) litter level.

For the evaluation of potential residues in females of childbearing age, a NOEL was chosen from a rat dietary two-generation reproductive toxicity study. At the LOEL (1,000 ppm), developmental toxicity was observed without any apparent maternal effects. However, the prolonged dosing regimen employed in such studies is often considered more appropriate for assessing chronic toxicity, which is generally associated with lower NOEL values, than acute toxicity. It is therefore possible that the choice of NOEL (500 ppm) may be overly conservative for assessing acute toxicity. However, this study provided the basis for listing resmethrin under California's Proposition 65, owing to developmental toxicity in the absence of apparent maternal toxicity[4]. It may therefore be most appropriate to consider the risk of developmental toxicity in exposed individuals from the perspective of likely exposure and absorbed dosage.

There are a number of uncertainties associated with the theoretical dietary exposure assessment. The theoretical commodity residue estimates (3 ppm for most RACs) may be highly overestimated. Based on use rates and application patterns for other registered pyrethroids with existing RAC tolerances, it is likely that the resmethrin theoretical commodity residues are overestimates. Additionally, while all possible above-ground harvestable RACs in California are included in the dietary food file, it is highly unlikely that every county in California would elect to conduct mosquito vector control simultaneously using resmethrin during the 30-day window allowed by the proposed Section 18. Moreover, it is unlikely that the spraying would occur over all crops and result in residues at harvest.

Recently, a comparative risk analysis of WNV illness vs. mosquito-control pesticide toxicity was conducted[29]. Both acute and subchronic risks were evaluated for resmethrin using a NOEL of 10 mg/kg/day for increased liver weight in a 6-month dog study[30]. Human health risks for urban residents were evaluated by assuming that tomatoes were contaminated at 0.105 ppm (mg/kg) resmethrin after ground-based spraying at 0.0078 kg/ha. The acute risk quotients (RQs) were well below 0.1 for all subpopulations studied using DEEM-FCID.™ This is equivalent to MOS values above 1,000. For subchronic exposure (assuming tomato residues of 0.012 ppm), which could be equated with seasonal exposure, RQs were all below 0.01 (i.e., MOS > 10,000). It was also pointed out that the delivery of pesticides by air resulted in lower concentrations at ground level, i.e., on crops, than in modeled truck-mounted applicators[29].

The theoretical residue estimates used here to calculate the dietary exposure from resmethrin use on agronomic crops resulted in MOS > 180 for all of the general population groups (male adults, seniors of both sexes, infants, and children). In addition, the commodity residue estimates resulted in a MOS of 520 for females of childbearing age when the NOEL of the 25 mg/kg/day was used to calculate dietary exposure. These calculations are considered unrefined estimates of potential and/or theoretical dietary exposure for the requested public health Section 18 emergency exemption. The calculated MOS values appear to be adequate to protect public health.
